# A Report of the Operative Treatment of Several Cases of Frontal and Maxillary Sinusitis1Read at the Surgical Section, Academy of Medicine, October 3, 1899.

**Published:** 1899-11

**Authors:** Frank Whitehill Hinkel

**Affiliations:** Buffalo, N. Y., Clinical professor of laryngology, University of Buffalo


### A REPORT OF THE OPERATIVE TREATMENT OF SEVERAL CASES OF FRONTAL AND MAXILLARY SINUSITIS.[1]

By FRANK WHITEHILL HINKEL, M. D., Buffalo, N. Y.,
Clinical professor of laryngology, University of Buffalo.

IN THE past few years the surgery of the sinuses adjacent to the nose and ears has transcended in interest the well-established methods of treatment of these organs themselves. Our ability to recognise diseased conditions of these sinuses has been increased, and by various modifications of technique our operative measures for their relief have been made more effective.

As you know, chronic empyema of the frontal sinus has little or no tendency to self cure. The narrow infundibulum, choked with swollen mucous membrane and granulations, affords very imperfect drainage to the products of an infective inflammation that may have extended from the nose. The dull frontal pain, the benumbed mentality, the depression of the general health, and the offensive unilateral nasal discharge, may improve at intervals, but only to be followed by fresh exacerbations; and erosion of the posterior wall of the sinus with fatal involvement of the cranial contents may occur at any time, though more often the ocular orbit is invaded. The case of frontal empyema, to which, with comments on its course and treatment, I would call your attention, is as follows :

Case I.—Mr. B., aged 38, was attacked by epidemic influenza in February, 1898, with severe pain over the left eye, followed by free purulent left-sided nasal discharge, at times of an offensive odor. For a number of years he had been subject to hay-fever symptoms in the summer time, with occasionally wheezy breathing, especially if in the country. When first examined in September, 1898, seven

---

1. Read at the Surgical Section, Academy of Medicine, October 3, 1899.

months after his influenza, there was free offensive purulent discharge from the left nostril. A unilateral discharge of muco-pus from the nose is indicative of one of three conditions : a foreign body lodged therein, or an osteitis, usually syphilitic, or an empyema of one of the accessory sinuses. In this case the first two conditions were excluded. There were present left supraorbital pains, and tenderness at times on pressure upon the left eyeball. Supraorbital pain is often present in antral disease without involvement of the frontal sinus, and this symptom alone has little significance as regards the diagnosis of frontal empyema. The general health was somewhat impaired.

Examination showed some crusts and muco-pus beneath the left middle turbinate in the vicinity of the orifice of communication between the antrum and the nasal chamber. There was slight tenderness on pressure over the left antrum and left frontal sinus. Antral translumination showed relative darkness of the left malar region. Translumination, when the difference in translucency of the two sides is pronounced, is of much value in confirming the diagnosis of antral empyema, but, as is often the case, if there be a general thickening of the nasal mucous membrane from long standing inflammation, its results may be so indecisive as to have little value. An imbedded root, the remains of the left superior second bicuspid tooth, which had been lost years before, was extracted and found sound and not penetrating the antrum. The antral floor was broken through and a large amount of very offensive pus syringed from the antrum. This treatment was kept up for a month by the patient at his home at a distance from Buffalo. At the end of this time there was very little flocculent discharge, without offensive odor, when the antrum was syringed, but pus was seen to drain from the extreme anterior portion of the left middle meatus immediately after the antrum was syringed clear. The anterior extremity of the left middle turbinate was then removed and the anterior ethmoid cells opened and curetted. In another month, October 20th, the patient reported with an increase of discharge from the left nasal chamber, and occasional left supraorbital pain, followed usually by increased nasal discharge. There was slight tenderness on firm pressure over the left frontal sinus. Frontal translumination was excellent on the right side and revealed a large sinus. There was darkness over the left supraorbital region. The left antrum contained a large amount of pus and presented the usual signs of antral empyema.

Opening of the frontal sinus was now recommended. In January, 1899, the patient, who had lost weight and whose general health was beginning to fail, consented to the operation. On January 25th, at the Buffalo General Hospital, after shaving the left eyebrow, an incision was made along the inner half of the left supraorbital ridge to a little beyond the middle of the glabella, the soft parts and periosteum were retracted and the anterior wall of the left frontal sinus opened with a small trephine. The incision thus made has cosmetic advantages over the vertical incision in the median line of the forehead. The regrowth of the eyebrow almost conceals the scar if the external wound heal kindly after the operation. The sinus was found filled with a greenish offensive pus. The incised tissues were protected by a dam of iodoform gauze and the sinus flushed with a normal salt solution until the pus no longer welled up. The sinus was unusually large, extending far externally and posteriorly over the left orbit. Its walls were thoroughly curetted and the naso-frontal canal, located by a curved probe, was enlarged with a trocar and curette until an ample opening communicated with the nasal chamber, presumably through the anterior ethmoidal cells. A strip of iodoform gauze instead of a drainage tube was introduced into the nasal chamber through the enlarged infundibulum, as suggested by Bryan, and the external incision closed by silk suture. A dressing of aseptic gauze and cotton was applied and retained by a gauze bandage. Intranasal insufflations of iodoform were applied every three hours, after gentle spraying of the nose with normal salt solution. No other treatment was given. There was no rise of temperature. The gauze drain was removed by the nostril on the third day. On January 30th, the sixth day, the wound was redressed and the stitches were removed. There was good union. On February 1st, the eighth day, the patient was discharged from the hospital. The left antrum on syringing was found entirely clear and no subsequent unusual discharge occurred from the left nasal chamber. There has been no return of symptoms of disease. There remains no specially noticeable scar and a gain of seventeen pounds in weight was reported some months since.

The points of interest in this case history are the masking of the primary frontal empyema by the symptoms and signs of the secondary antral abscess; the persistence of the antral discharge despite drainage and cleansing of the antrum, and its immediate cessation as soon as drainage of the frontal sinus was secured. Bryan has demonstrated anatomically that occasionally the infundibulum by a peculiar conformation of the external wall of the nose drains its discharges into the corresponding antrum rather than into the anterior superior portion of the nasal chamber. This case appears to be of that type and shows the difficulty at times of locating the source of a purulent nasal discharge.

You are familiar with the procedure usually followed for the relief of antral empyema. The purpose of operative interference is, first, to remove if possible the exciting cause, and, second, to secure drainage and a counter opening for cleansing. The peculiar location of the antral orifice in the upper third of the nasal wall of the antrum prevents the free drainage of pathological accumulations within the antrum through the normal passage. To secure the required drainage it has long been the custom to extract the second bicuspid or first molar tooth on the affected side. The roots of these teeth frequently penetrate the bony floor of the antrum and access for small instruments is easy through the opening left by their extraction. Should the bony floor of the antrum be intact, it is very thin above the socket of the extracted tooth and easily perforated.

A large proportion of cases of empyema of the antrum is due to primary dental disease, particularly if involving the bicuspids or molars. The operation just described secures at once the removal of the cause of disease and the required counter opening for cleansing. In comparatively recent cases of dental origin it unquestionably best fulfils the indications for treatment. Where the teeth are not diseased and the antral involvement is comparatively recent, drainage may be secured and a cure accomplished without the sacrifice of a sound tooth by the method suggested by Mickulicz. The nasal wall of the antrum is perforated beneath the inferior turbinate and the antrum washed out at intervals through a short canula that may be left *in situ* without inconvenience to the patient. This method is applicable to the treatment of a limited number of cases as well as for diagnostic purposes as the procedure is not formidable.

In cases where the antral empyema is of long standing, whether of dental or nasal origin, the operations described are usually insufficient for a cure. Free access must be had to the antral cavity that its inflamed thickened and granulated mucous membrane may be thoroughly curetted and subjected to subsequent treatment if necessary. A sufficient opening of easy access can be made only in the anterior wall of the antrum in the region of the canine fossa, any diseased teeth being extracted at the same time as a preventive measure. A drainage tube is generally inserted through which subsequent treatment can be continued. Some operators repeatedly tampon the cavity of the antrum for a long period, but I do not see any advantage to accrue from this, as it is impossible to obliterate by connective tissue formation this large cavity between fixed bony walls, and I can conceive of no other purpose to be gained by the tamponing. The results of this more radical method have not always been entirely satisfactory. In many cases the obstinate antral discharge continues unabated if less offensive. The drainage tube itself is irritating and if the opening through the canine fossa be kept patulous for treatment, it is a ready means of infection from the bacterial hot bed of the mouth. About two years since Luc suggested a modification of the radical antral operation. Caldwell, of England, had proposed the same thing a few years earlier, but his suggestion failed to secure attention. Luc, after the antrum was opened through the canine fossa, made a counter opening through the nasal wall beneath the inferior turbinate and then closed the opening through the canine fossa by stitches through the mucous membrane of the oral conjunctiva.

I wish to report two operations for chronic antral empyema, in which I followed the method of Luc. Each of these cases was of great chronicity. In each case I followed the usual steps for laying bare and opening the anterior wall of the antrum, and I will confine the description to the novel features of the operation; the opening of the nasal wall of the antrum beneath the inferior turbinate, and the immediate closure of the gingivo-labial incision. I found the free hemorrhage following the incision into the gingivo-labial conjunctiva lessened by an injection of a 1 per cent. cocaine solution beneath the mucous membrane just as the anesthetic was about to be administered. In one case in which I had been treating the antrum through Freeman's canula beneath the inferior turbinate, I was able to reduce the hemorrhage, that is so profuse when the antrum is opened, by injecting into the antrum just before the operation about a drachm of a solution of suprarenal gland.

Case I.—Miss S., aged 37, empyema of left antrum, marked by moderate discharge and at intervals deep-seated pain in the malar region. On February 9, 1899, at the Buffalo General Hospital, with trephine and bone forceps I removed part of the anterior wall of the left antrum. There was no particular difficulty in breaking down the nasal wall beneath the inferior turbinate. I passed a curved trocar within the nostril and beneath the anterior extremity of the inferior turbinate and readily broke down the thin antral wall. Using the point of the trocar as a guide through the freely welling blood within the antrum, I enlarged the opening with a sharp spoon. I encountered great difficulty in endeavoring to pass an iodoform gauze drain from the antrum through the nasal chamber and out at the nostril. I closed the gingivo-labial incision with silk suture. The escape of air through this aperture for several days, when the nose was blown, showed that the closure was imperfect. However, by the fifth day, when the stitches were removed, the incision was closed. A small area of numbness about the left angle of the mouth was noticed for several days after the operation. After the fifth day the antrum was flushed by means of a Eustachian catheter through the opening in the nasal wall. A slight amount of muco-pus escaped at each washing. After several weeks the nasal opening contracted so that a small canula could not be introduced and there was some return of pain. Under cocaine I cut away the anterior dependent portion of the inferior turbinated bone and with a sharp spoon enlarged the opening into the nasal wall of the antrum. This procedure was followed by relief and on April 27th the patient was discharged apparently well.

Case II.—Miss M., left antral empyema, operated on at the Buffalo General Hospital, March 9, 1899. I had previously been treating the case without relief by cleansing through a perforation in the nasal wall of the antrum. The inferior turbinate was dependent and lay close to the antral wall. I removed the anterior portion of it to facilitate the passage of the trocar into the antrum. The antral walls were carefully curetted. I did not introduce a drainage tube into the nose. After a counter opening had been made beneath the inferior turbinate the gingivo-labial incision was allowed to fall into place without stitches and union followed nicely in a few days. However, a small abscess occurred later on the margin of the wound, requiring curetting. It did not communicate with the antrum. This case improved rapidly for several weeks. The antrum was readily flushed through the nasal opening and I found injections of alcohol serviceable in reducing the discharge and lessening its purulent character. In about three weeks the nasal opening began to grow small and the discharge to be somewhat confined within the antrum. On April 19th, I enlarged the nasal opening under cocaine with a curette and used at intervals injections of alcohol and of a solution of iodoform in ether. Since May 26 there has been no unusual nasal discharge nor any symptoms of antral disease. Antral translumination is equally good, though there is no left pupil glow. This case therefore may be considered cured.

Valid conclusions as to the value of this operation cannot be drawn from so limited a number of cases, but I offer their details for your consideration. The limitations of the operation and the proper choice of cases for its performance will be facilitated by reports of its results whether successful or otherwise. I see several directions in which my own technique can be improved in subsequent operations. The introduction of the drainage tube I have found the most difficult step in the operation. To facilitate this I have had made for me a modification on a small scale of Belocq's canula. Introduced through the nose and with the probe point thrust forward and upward into the antrum, it brings readily into reach the ligature to which the drainage tube or strip of gauze can be attached and then drawn through the opening in the nasal wall and out at the nostril.

The suturing of the gingivo-labial incision seems from my limited experience to be unnecessary. It is difficult to keep the stitches already inserted from being somewhat torn out during the insertion of the later stitches, on account of the manner in which the parts must be drawn upon to secure access to the lips of the wound. The parts coapt readily without stitches and there is but little necessary motion of the gingivo-labial fold. The wound need not be disturbed if the patient be fed upon soft food, taking care to use the side of the mouth opposite to the operation in masticating and to avoid blowing the nose violently. As shown by my second case healing occurs readily without stitches if a little care is used.

412 FRANKLIN STREET.

DISCUSSION.

Dr. PARK LEWIS thought that a less radical method of surgical procedure might be followed by better results.

DR. PARK recalled operating several years ago in antral cases by the method advocated by Mikulicz. He advises the most radical methods in all old intractable inflammatory infections of the sinuses or antrum. In opening into the antrum the opening in the anterior wall should be large enough to admit one or two fingers and to permit of inspection as well as digital examination.

Dr. BLAAUW reported two cases of chronic frontal sinusitis in which he chipped off part of the anterior wall after a vertical incision and in each case recovery was rapid.

DR. MILLINER reported an anatomical anomaly of the antrum in the first case of chronic antral disease that he had operated upon. A bony septum dividing the antrum into two cavities was discovered and the unaffected cavity was the first to be opened into. A continuation of the opening led into the second cavity where the infection was found.

